# A sequence-specific DNA binding small molecule triggers the release of immunogenic signals and phagocytosis in a model of B-cell lymphoma

**DOI:** 10.1017/S0033583515000104

**Published:** 2015-11

**Authors:** JeenJoo S. Kang, Peter B. Dervan

**Affiliations:** Division of Chemistry and Chemical Engineering, California Institute of Technology, Pasadena, CA 91125, USA

**Keywords:** Py-Im Polyamide, damage-associated molecular patterns, necrotic cell death, immunogenic cell death, DNA-binding small molecule, telomere sequence

## Abstract

Means to cause an immunogenic cell death could lead to significant insight into how cancer escapes immune control. In this study, we screened a library of five pyrrole–imidazole polyamides coding for different DNA sequences in a model of B-cell lymphoma for the upregulation of surface calreticulin, a pro-phagocytosis signal implicated in immunogenic cell death. We found that hairpin polyamide **1** triggers the release of the damage-associated molecular patterns calreticulin, ATP and HMGB1 in a slow necrotic-type cell death. Consistent with this signaling, we observed an increase in the rate of phagocytosis by macrophages after the cancer cells were exposed to polyamide **1**. The DNA sequence preference of polyamide **1** is 5′-WGGGTW-3′ (where *W* = A/T), indicated by the pairing rules and confirmed by the Bind-n-Seq method. The close correspondence of this sequence with the telomere-repeat sequence suggests a potential mechanism of action through ligand binding at the telomere. This study reveals a chemical means to trigger an inflammatory necrotic cell death in cancer cells.

## Introduction

Avoidance of immune destruction has been called the seventh hallmark of cancer (Dunn *et al.* 2004a; Hanahan & Weinberg, 2011). According to the immuno-editing paradigm, the immune system recognizes and destroys those proto-oncogenic lesions capable of triggering an immune response, while those that escape immune control grow to become clinically detectable disease (Dunn *et al.* 2004b; Koebel *et al.* 2007; Schreiber *et al.* 2011; O’Sullivan *et al.* 2012). Studies suggest that therapies that enlist the immune system maintain more durable disease control in the clinical setting (Eggermont *et al.* 2014). Chemical methods to cause immunogenicity in cancer cells would be an important tool toward understanding immunomodulation in the treatment of cancer. A prerequisite for the activation of an anti-cancer immune response is the recognition of the damaged cells as a threat. Damaged cells release immunostimulatory molecules, called damage-associated molecular patterns (DAMPs), to recruit and activate professional phagocytes such as macrophages and dendritic cells (Matzinger, 2002; Obeid *et al.* 2007; Jaiswal *et al.* 2010). These antigen-presenting cells engulf and process the cancer cells to further prime the immune system for targeted elimination of cancer (Tseng *et al.* 2013).

Although most chemotherapeutic regimens cause a non-immunogenic or even tolerogenic cell death, recent reports suggest anthracyclins or *γ*-radiation are particularly effective because they result in the release of DAMPs (Obeid *et al.* 2007; Zitvogel *et al.* 2008). The extracellular exposure of the intracellularly abundant molecules calreticulin (CRT), HMGB1 and ATP have been suggested to form a spatiotemporal code for immunogenicity (Zitvogel *et al.* 2010; Kepp *et al.* 2011). The presentation of CRT, an abundant ER-resident chaperone protein, to the cell surface was identified as a necessary and sufficient pro-phagocytic signal for professional phagocytes (Obeid *et al.* 2007). The study showed that stimulation of CRT surface expression by anthracyclins or adsorbtion of the calreticulin protein on the cell surface was sufficient to elicit an anti-cancer immune response in syngeneic mice (Obeid *et al.* 2007). Weissman and co-workers further demonstrated in the Raji cell line, a model of human B-cell non-Hodgkin’s lymphoma, that CRT is the dominant pro-phagocytosis signal which is necessary for engulfment by human macrophages (Chao *et al.* 2010). Furthermore, ATP released from the cytosol into the local microenvironment serves as a lymphocyte recruiting and activating chemokine (Idzko *et al.* 2002; Aymeric *et al.* 2010). Lastly, the nucleus-resident protein HMGB1 can be secreted into the surroundings as an inflammatory adjuvant and was shown to be necessary for a durable anti-cancer response in mice (Rovere-Querini *et al.* 2004; Guerriero *et al.* 2011). Identification of additional small molecules that trigger the release of these DAMPs from tumor cells would be of utility to the field in addressing the heterogeneity of cancers. We became interested in expanding the examined chemical space for compounds capable of causing an immunogenic cell death. Because the DNA damage pathway has been implicated in immunogenic signaling (Gasser *et al.* 2005) and anthracyclins are DNA-intercalating ligands, we sought to explore a class of minor groove DNA-binding oligomers hitherto not studied for this biological activity.

**Fig. 1:**
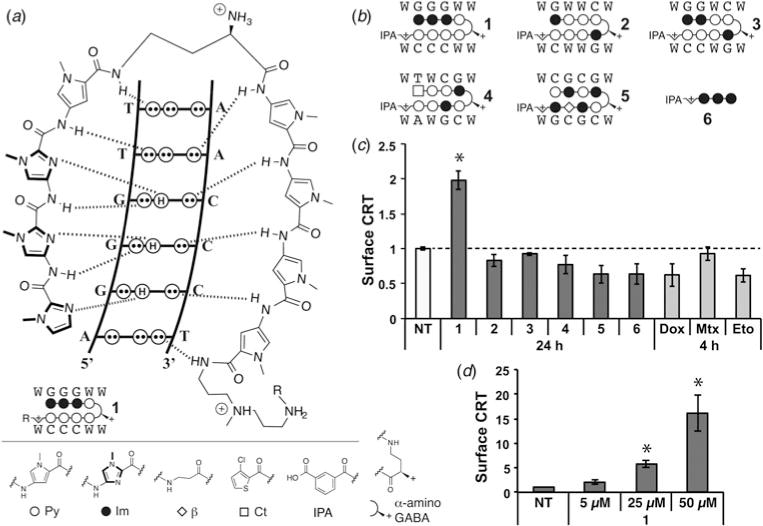
Screen of Py–Im polyamide library **1–6** for stimulation of surface calreticulin (CRT) in Raji cells. (*a*) Py–Im polyamide recognition of DNA minor groove and corresponding ball-and-stick notation. (*b*) Structures of compounds in polyamide library. (*c*) Surface expression of calreticulin measured by flow cytometry, median fluorescence normalized to non-treated control (NT). Cytotoxic controls doxorubicin (**Dox**, 5 μM), mitoxantrone (**Mtx**, 1 μM) and etoposide (**Eto**, 30 μM). (*d*) Dose dependence of cell surface CRT to polyamide **1** was measured by flow cytometry after 24 h treatment. Measurement and error bars of the standard deviation are in triplicate and representative of two independent experiments; asterisk indicates *p* < 0.05 by two-tailed Student’s *t* test compared with NT.

Hairpin pyrrole–imidazole (Py–Im) polyamides are a class of sequence-specific oligomers that bind in the minor groove of DNA (Wade *et al.* 1992; Trauger *et al.* 1996; Kielkopf *et al.* 1998a, b; White *et al.* 1997, 1998). Sequence preference is achieved by the pair-wise, co-facial arrangement of aromatic amino acids that distinguish the edges of the four Watson–Crick base pairs as shown in Fig. 1a (Dervan & Edelson, 2003). Pairing rules for programmable specificity have been established: Im/Py specifies a G•C base pair, Hp/Py codes for T•A base pairs and Py/Py binds both T•A/A•T (White *et al.* 1998). Eight-ring hairpin polyamides are linked in an antiparallel fashion by a central aliphatic *γ*-aminobutyric acid unit (Mrksich *et al.* 1994). Polyamides of this hairpin architecture have affinities for match sites similar in magnitude to natural DNA-binding proteins, with *K*_a_’s of 10^8^–10^10^ M^−1^ (Hsu *et al.* 2007). Eight-ring hairpins of this class are cell-permeable and modulate transcription in both cells and mice (Nickols *et al.* 2007; Nickols & Dervan, 2007; Raskatov *et al.* 2012; Yang *et al.* 2013). In this study, we screened a small library of Py–Im polyamides coding for different six base pair DNA sequences in Raji cells for the upregulation of surface calreticulin. We found one hairpin polyamide which displayed activity in this screen and characterize its potential for causing an immunogenic cell death.

## Materials and methods

### Chemicals and reagents

All chemicals were purchased from Sigma-Aldrich unless otherwise noted. Py–Im polyamides were synthesized by microwave-assisted, solid-phase synthesis on Kaiser oxime resin (Novabiochem) according to previously described protocols (Baird & Dervan, 1996; Puckett *et al.* 2012). Polyamide **5** was synthesized on hydrazine resin (855037, Novabiochem) in the same manner as the other polyamides and cleaved from resin in the same manner after 10 min oxidation of the hydrazine by Cu(II)SO_4_ in pyridine and DMF. Polyamides were purified by reverse-phase HPLC and lyophilized. EZ-Link NHS-PEG4-biotin (Pierce) was conjugated in 5% Hünig’s base in DMF. Purity and identity of compounds were verified by analytical HPLC and matrix-assisted laser desorption/ionization time-of-flight mass spectrometry (SI Table 2).

Brefeldin A was purchased from BD Biosciences, 7-AAD from eBiosciences and Z-VAD-fmk from Promega. Antibodies purchased from Abcam are: polyclonal rabbit anti-calreticulin (ab2907), polyclonal rabbit anti-ERp57 (ab10287), polyclonal goat anti-rabbit Alexa Fluor 488 (ab150081). Antibodies from Life Technologies are: anti-Annexin V Alexa Fluor 488 conjugate (A13201) and mouse monoclonal anti-actin (AM4302). Antibodies from Cell Signaling Technologies are: rabbit polyclonal anti-PARP (9542) and rabbit monoclonal anti-phospho-H2AX (ser139, 9718).

### Cell culture

Raji cells (ATCC) were maintained in RPMI media (Life Technologies) with 10% fetal bovine serum (FBS, Omega Scientific) and 5 mM glutamine (Life Technologies). K562 cells (ATCC) were cultured in IMDM (ATCC) media and 10% FBS. A549 and PC3 cells (ATCC) were maintained in Kaign’s modified F-12 K media (Gibco) supplemented with 10% FBS and 5 mM glutamine. Peripheral blood macrophages (Stemcell Technologies) were thawed and plated in DMEM (ATCC) and supplemented with 5 mM glutamine and 10% FBS for at least two days and no longer than 6 days before use.

### Flow cytometry

Raji and K562 cells were plated at 1 × 10^5^ cells ml^−1^ in 96-well plates at 100 μl and treated immediately after plating. A549 and PC3 cells were plated in 24-well plates at 6 × 10^4^ cells ml^−1^ at 400 μl and were allowed to adhere overnight prior to treatment. Adherent cells were harvested with Accutase (Life Technologies).

For the CRT and ERp57 detection experiments, harvested cells were washed with cold staining buffer: HBSS (Life Technologies) with 2.5 mg ml^−1^ BSA (Amresco), 10 mM HEPES (Life Technologies), 0.1 mM MgCl_2_ (Life Technologies) and DNAse (Roche). Cells were incubated with human FcR block (eBiosciences) and primary antibody in cold staining buffer. This was followed by washing and incubation with dye-conjugated anti-rabbit secondary antibody. 7-AAD, forward- and side-scatter were used to gate for live cells. Secondary antibody alone was used as a control (SI Fig. 2). The median fluorescence intensity of live cells is normalized to non-treatment. Data were acquired on the FACS Calibur and analyzed with Flowjo software. Measurements and standard deviations are in triplicate and representative of at least two independent experiments; asterisk indicates *p* < 0.05 by two-tailed Student’s *t* test compared with non-treatment.

For cell death analysis flow cytometry experiments, Raji cells were harvested after treatment and washed with cold staining buffer and incubated with FcR block and dye-conjugated anti-Annexin V. Cells were washed with staining buffer and taken for immediate analysis. 7-AAD was added to samples maintained at 4 °C and data was acquired soon thereafter on the MACS VYB. Data was acquired in triplicate from two independent experiments and analyzed with Flowjo (SI Tables 5 and 6).

### Bind-n-Seq of polyamide 1b

Highest affinity DNA binding sites of polyamide–biotin conjugate **1b** were determined according to previously reported methods (Meier *et al.* 2012). In brief, polyamide **1b** was equilibrated at 25 or 250 nM overnight in a DNA library of all possible 21 mers. DNA bound to **1b** was affinity purified with streptavidin magnetic beads (M-280 Dynabeads, Life Technologies) and eluted. Isolated DNA was amplified by touchdown PCR and submitted for sequencing on an Illumina HiSeq 2000 Genome Analyzer. The MERMADE script was used to distribute data by bar-code and a fasta file of a random 25% of sequences was generated for DREME motif analysis. Data are representative of three independent experiments (SI Fig. 4).

### DNA thermal denaturation assay

DNA of the sequence 5′-CTTAGGGTTAGC-3′ and its complement were purchased from Integrated DNA Technologies and annealed. The oligonucleotides were mixed with hairpin polyamide **1** to a final concentration of 2 and 3 μ M, respectively, in 1 mL total volume. An aqueous solution of 10 mM sodium cacodylate, 10 mM KCl, 10 mM MgCl_2_ and 5 mM CaCl_2_ at pH 7.0 was used as analysis buffer. The assay utilized a Varian Cary 100 spectrophotometer to heat samples to 90 °C, cool to a starting temperature of 25 °C, and then heat at a rate of 0.5 °C min^−1^ to 90 °C. Denaturation profiles were recorded at *λ* = 260 nm and melting temperatures were detected by the maximum of the first derivative. Data show the combined mean and standard deviation of triplicate measurements from two independent experiments.

### Immunoblot assays

Raji cells were plated at 10^5^ cells ml^−1^ in 10 cm diameter dishes and dosed with the indicated treatment. Cells were washed with cold PBS and lysed for 10 min in lysis buffer (50 mM Tris–HCl pH 7.4, 1 mM EDTA, 150 mM NaCl, 1% Triton X-100) containing protease inhibitors (Complete, Roche), 1 mM PMSF and phosphatase inhibitors. Samples were clarified by centrifugation at 14 000 × ***g*** for 10 min, quantified with Bradford reagent (Bio-rad), denatured by boiling in Laemmli buffer (LI-COR) for 5 min and separated by sodium dodecyl sulfate–polyacrylamide gel electrophoresis using AnyKD gradient gels (Biorad). Protein was transferred to a PVDF membrane (Millipore) and blocked with Odyssey blocking buffer (LI-COR). Both primary antibodies and appropriate IR-dye conjugated secondary antibodies (LI-COR) were incubated in blocking buffer with 0.2% Tween. Anti-actin was used to control for equal loading and experiments were done in at least two independent biological replicates. Bands were visualized on a LI-COR Odyssey infrared imager.

### ATP bioluminescence assay

Raji cells were plated into 96-well cell culture plates at 200 μl per well and 10^5^ cells ml^−1^, in quadruplicate per condition. After the indicated treatment, media and cells were transferred to a 96-well PCR plate and centrifuged at 130 × ***g*** for 5 min. The supernatant was collected for analysis of ATP content using an ATP bioluminescence kit (FLAA, Sigma). The assay was performed according to the manufacturer’s protocol and luminescence was measured using a Flexstation 3. Measurements and standard deviation are technical quadruplicate and biological triplicate. Asterisk indicates *p* < 0.05 by two-tailed Student’s *t* test compared with non-treatment.

### HMGB1 ELISA

Raji cells were plated into 96-well cell culture plates at 100 μl per well, 10^5^ cells ml^−1^, in duplicate. Cells were treated with polyamide **1** and **2** as indicated and the supernatant collected. HMGB1 was measured using the Shino-Test ELISA kit (IBL international) according to the manufacturer’s instructions on the Flexstation 3. Measurement and standard deviations were determined from technical duplicate from two independent replicates. Asterisk indicates *p* < 0.05 by two-tailed Student’s *t* test compared with non-treatment.

### Caspase luciferase assay

Raji cells were plated into 96-well cell culture plates at 100 μl per well and 10^5^ cells ml^−1^, in triplicate per condition. Media was included as a blank control. After the indicated treatment, media and cells were transferred to an opaque white 96-well plate containing 100 μl in each well of the caspase-dependent luciferase reagent, prepared as per the manufacturer’s instructions (Caspase-Glo 3/7, Promega). The mixture was left at room temperature for 1 h prior to measurement on the Flexstation 3 (Molecular Devices). Measurements and standard deviations were determined in triplicate and done in biological duplicate.

### Metabolic activity assay

Raji cells were plated into 96-well clear bottom cell culture plates at 100 μl per well and 10^5^ cells ml^−1^, in quadruplicate per indicated condition. Media was used as a background control. Metabolic activity was assessed using the WST-1 reagent (Roche) as per the manufacturer’s instructions. Measurements and standard deviations were determined in quadruplicate and done in biological duplicate.

### Phagocytosis assay

Peripheral blood macrophages were plated at 2 × 10^4^ cells per well in 24- or 48-well plates in DMEM. Immediately prior to use, macrophages were washed with HBSS and stained with carboxyfluorescein diacetate succinimidyl ester (CFSE, eBioscience) in HBSS for 5 min. Macrophages were washed of excess dye and returned to DMEM for incubation. Target Raji cells were plated in 96-well plates at 1 × 10^5^ cells ml^−1^ in 200 μl of RPMI and treated as described. Target A549 cells were plated in 24-well plates at 2 × 10^4^ cells per well 15 h before beginning treatment. After treatment, cells were harvested with Accutase if necessary and washed with HBSS and incubated with pHrodo succinimidyl ester (Life Technologies) diluted to 2 μM in HBSS for 5 min. Cells were then washed by centrifugation at 150 × ***g***, re-suspended in DMEM, counted on a Biorad TC10 cell counter, and 5 × 10^4^ cells per well were added to macrophages. After incubation for 2.5 h at 37 °C, media and non-adherent cells in each sample were aspirated and saved. Adherent cells were trypsinized, aspirated and combined with saved media mixture. Cells were washed once with PBS and fixed in 1% formaldehyde and kept at 4 °C until assessment on the MACS VYB flow cytometer. The percentage of phagocytosis was calculated as the percentage of double positive cells among fluorescein+ macrophages. Measurements and standard deviations are taken from three independent experiments and asterisks indicate *p* < 0.05 by two-tailed Student’s *t* test compared with non-treatment.

### Confocal microscopy

For images of phagocytosis, cells treated in the manner described above were put on 35 mM glass-bottom dishes (MatTek). Confocal images were acquired using a 40× oil immersion objective on a Zeiss LSM 5 Exciter microscope.

## Results

### Polyamide 1 upregulates calreticulin on the cell surface

We tested five Py–Im polyamides (**1–5**, full structures in SI Fig. 1) that bind five unique DNA sequences (5′-WGGGWW-3′, 5′-WGWWCW-3′, 5′-WGGWCW-3′, 5′-WTWCGW-3′ and 5′-WCGCGW-3′, respectively, where *W* = A/T) and have demonstrated biological activity (Fig. 1b) (Swalley *et al.* 1996; Nickols & Dervan, 2007; Nickols *et al.* 2007, 2013; Kang *et al.* 2014). Raji cells, which have previously been utilized in CRT, phagocytosis, and immunotherapy animal models, were dosed at 5 μM for 24 h with each of the polyamides **1–5**. In addition, 5 μM doxorubicin (**Dox**) and mitoxantrone (**Mtx**, 1 μM) were included as representative anthracyclins. The topoisomerase inhibitor etoposide (**Eto**, 30 μM) was also included for comparison. A 4 h exposure time point was used to measure CRT due to the high toxicity of these chemotherapeutics and to be in the range of literature precedent (Obeid *et al.* 2007). We measured surface CRT by flow cytometry in a population gated for live cells and saw a statistically significant, twofold increase in cells treated with only polyamide **1** (Fig. 1c). In contrast, we saw no activity with the other polyamides **2–5** despite their structural similarity. Notably, polyamide **2** has been most extensively studied among this class and has shown activity in prostate cancer xenograft models and DNA damage response (Yang *et al.* 2013; Martínez *et al.* 2015). Remarkably, polyamides **3** and **1** share the same molar weight and composition of Im/Py pairs, and only differ by the exchange of one Im/Py ring pair. To assess the structure activity relationship of the imidazole trimer portion of the hairpin oligomer **1**, we synthesized and examined ImImIm polyamide **6**. This was tested because imidazoles are known to complex with calcium and CRT is involved in calcium homeostasis (Michalak *et al.* 2009). We did not, however, observe an increase of CRT with polyamide **6**, which suggests the imidazole trimer alone is not sufficient to trigger the surface expression of CRT. We observed no increase in CRT with the two anthracyclins tested, **Dox** and **Mtx**, or with **Eto**. The diminished response to anthracyclin treatment in Raji cells, as compared with that reported in the literature with murine colon cancer cells, reflects a known range of CRT response to anthracyclins and may be attributable to a difference in cell lines (Tufi *et al.* 2008; Peters & Raghavan, 2011). We next increased the treatment concentration of **1**–25 and 50 μM and found that CRT exposure increased in a dose-dependent manner (Fig. 1d, SI Fig. 2a). The results indicate polyamide **1** is unique in our small library of compounds in its modulation of surface expression of CRT.

### Polyamide 1 preferentially binds the sequence 5′-WGGGTW-3′

**Fig. 2:**
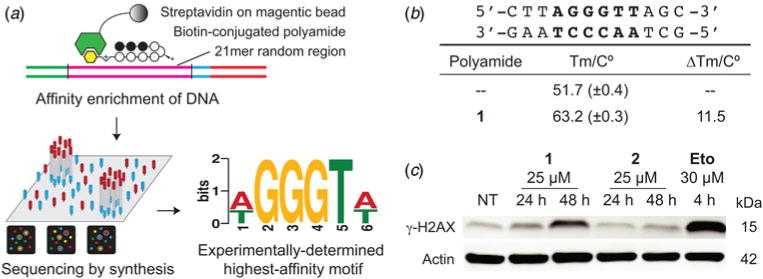
Preferred DNA-binding sequence of polyamide **1**. (*a*) Polyamide **1b** (SI Fig. 1b), the biotinylated analog of **1**, was tested in a Bind-n-Seq assay and found to preferentially bind the described DNA sequence motif. (b) Affinity of **1** was assessed by a DNA thermal stabilization assay against dsDNA containing the telomeric-repeat sequence 5′-TTAGGG-3′. (c) Immunoblot of *γ*-H2AX and actin control after the indicated treatment.

Py–Im polyamides are a class of sequence-specific DNA-binding ligands and the DNA-binding preferences of polyamide **1** may be related to a mechanism of action. By the pairing rules, polyamide **1** is a perfect match to 5′-WGGGWW-3′, not unlike the TTAGGG-repeat sequence found in human telomeres. Indeed, dye-conjugated tandem hairpin Py–Im polyamides that recognize 10 base pairs of this repeat sequence can be used to stain telomeres in permeabilized cells (Maeshima *et al.* 2001; Kawamoto *et al.* 2013). We sought to confirm the preferred binding sequence motif of **1** using the Bind-n-Seq method. Bind-n-Seq couples affinity enrichment with next-generation sequencing to query genome-sized sequence space for high-affinity binding sequences (Meier *et al.* 2012). We modified polyamide **1** at the C-terminal position with a biotin-label to afford **1b** (full structure in SI Fig. 1b) for submission to Bind-n-Seq. Polyamide **1b** strongly preferred binding the DNA sequence motif of 5′-WGGGTW-3′ (Fig. 2a, SI Table 4). We additionally confirmed the affinity of polyamide **1** for this sequence by measuring the thermal stabilization afforded to sequence-matched double-stranded DNA (Pilch *et al.* 1996). We tested a DNA fragment that included the telomeric DNA sequence 5′-TTAGGGTTAG-3′ (Fig. 2b). Polyamide **1** stabilized the 12 base pair DNA fragment by 11.5 °C, suggesting a high-affinity hairpin polyamide. Lastly, we detected a marker of DNA stress, phosphorylation of serine 139 on the histone H2AX, after treatment with polyamide **1** but not **2** (Fig. 2c). We chose the 25 μM dose for this experiment and others described below because it resulted in a robust sixfold increase in surface CRT. We posit that the CRT effect may be due to the unique properties of the DNA target sequence of polyamide **1** in cell biology. The DNA stress, thermal denaturation and Bind-n-Seq results together suggest the telomere sequence is a plausible target for the mechanism of action of polyamide **1**-mediated CRT exposure.

### Polyamide 1 triggers CRT in a different manner than do anthracyclins

**Fig. 3:**
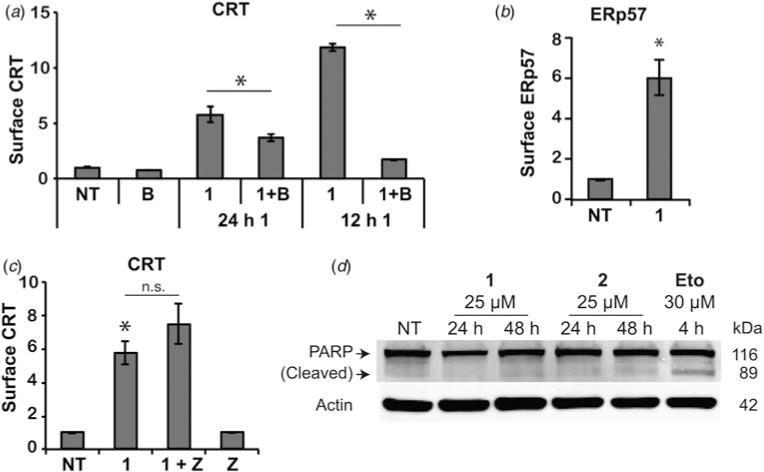
Treatment of Raji cells with polyamide **1** triggers anterograde CRT transport by a different mechanism than previously reported for anthracyclins. (*a*) Surface CRT was measured by flow cytometry after Raji cells were treated with 25 μM polyamide **1** for 12 or 24 h and Brefeldin A (**B**) for the final 12 h. (*b*) Co-chaperone ERp57 was measured on the cell surface by flow cytometry after 24 h treatment with **1** at 25 μM. (*c*) Caspase inhibitor Z-VAD-fmk (**Z**, 10 μM) and polyamide **1** (25 μM) were dosed together for 24 h and assessed by flow cytometry for surface CRT. (*d*) Immunoblots for PARP cleavage and actin control after the indicated treatments are shown. All flow cytometry analyses were done in triplicate and are representative of at least two independent experiments. Error bars show standard deviations and asterisks mark statistically significant changes (*p* < 0.05) by two-tailed Student’s *t* test compared with non-treatment (unless another comparator is marked).

To better understand the effects of polyamide **1**, we compared its trigger of CRT with reports of CRT induction by anthracyclins (Panaretakis *et al.* 2009). Anthracyclin-induced CRT exposure has been described to occur with the co-chaperone ERp57 by anterograde ER-Golgi transport (Panaretakis *et al.* 2009). To interrogate whether polyamide **1** induces CRT export to the cell surface by ER–Golgi transport, in Fig. 3a, we applied the Golgi transport inhibitor Brefeldin A (**B**) to Raji cells (Lippincott-Schwartz *et al.* 1989). Due to the toxicity of **B**, we could only dose **B** for 12 h. With 24 h treatment with polyamide **1** and 12 h treatment with **B**, we saw statistically significant reduction of CRT on the cell surface when compared with polyamide **1** treatment alone (Fig. 3a). When polyamide **1** and **B** were dosed together for 12 h, we saw near ablation of all polyamide **1**-induced CRT, demonstrating the necessity of this pathway for transport. This further suggests CRT has a half-life on the cell surface of at least 12 h. We additionally measured co-chaperone ERp57 by flow cytometry and found that after treatment with **1** for 24 h, ERp57 increased on the cell surface to a similar extent as did CRT, approximately sixfold (Fig. 3b). These results are consistent with previous reports that the translocation of these ER-resident chaperones to the plasma membrane occurs by anterograde ER–Golgi transport (Panaretakis *et al.* 2009).

**Fig. 4:**
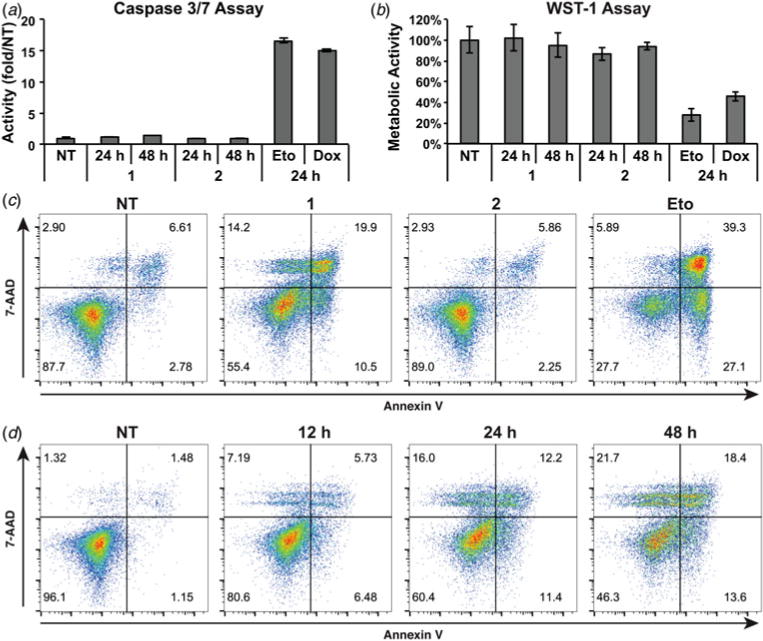
Raji cells undergo a slow, necrotic type cell death after treatment with polyamide **1**. (*a*) Caspase 3/7 activity was assessed by a luciferase assay after the indicated treatment. Measurement is representative of two independent experiments and error bars show standard deviations of triplicate measurement. (*b*) Cellular metabolism as a proxy for cytotoxicity was measured with a WST-1 assay. Cytotoxic controls etoposide (**Eto**, 30 μM) and doxorubicin (**Dox**, 5 μM) were included. Measurements were normalized to non-treatment. Graph is representative of two independent experiments and error bars represent standard deviations of technical quadruplicate. (*c*) Flow cytometry assessment of Raji cells treated with the indicated compounds for 24 h and stained for plasma membrane permeability (7-AAD) and phosphatidylserine exposure (Annexin V). Live: lower left; early necrotic: upper left; secondary necrotic: upper right; apoptotic: lower right. Representative plots shown of triplicate measurements from two independent experiments. (*d*) Assessment as in (*b*) after 12, 24 and 48 h exposure to polyamide **1** at 25 μM.

Anthracyclin-induced CRT has previously been reported to be mediated by a caspase 8- and caspase 3/7-dependent pathway that can be disrupted with the pan-caspase inhibitor Z-VAD-fmk (**Z**) (Panaretakis *et al.* 2009). Upon co-dosing polyamide **1** with **Z**, however, we saw no decrease in CRT exposure (Fig. 3c). This caspase-independence was unexpected in light of literature precedent as well as the cytotoxicity we observed of **1** during flow cytometry experiments. We confirmed the lack of PARP cleavage, a caspase substrate cleaved during apoptosis, by immunoblot (Fig. 3d). We then directly measured caspase activity with a luciferase assay after treatment with hairpin polyamides **1** and **2** as well as **Eto** and **Dox** (Fig. 4a). We measured a slight increase, approximately 1.2-fold, upon treatment with **1** for 24 h and no increase after treatment with 2. In contrast, we measured significant increases of approximately 15-fold each after treatment with Eto and Dox (Fig. 4a). The lack of caspase-dependence and caspase activation would suggest the trigger of CRT by **1** is different from anthracyclin DNA intercalators.

### Polyamide 1 induces a slow necrotic-type cell death

We measured cytotoxicity via the metabolic rate with the WST-1 reagent in a colorimetric assay. We were surprised to find that treatment with **1**, at both 24 and 48 h, had little effect on the bioreduction of WST-1 to the formazan dye, suggesting no diminution of metabolic rate (Fig. 4b). This is in stark contrast to the cytotoxic **Eto** and **Dox** which showed drastic reduction in cellular metabolism, consistent with the apoptotic program. This led us to assess whether polyamide **1** may direct the Raji cells toward programmed necrosis. Necrosis is an immunogenic, inflammatory type of cell death that is usually attributed to harsh physical insult such as freeze–thaw cycles. Recently, a biologically controlled necrosis program has been described where cells undergo early plasma membrane permeabilization with active inflammatory signaling and an oxidative burst (Kaczmarek *et al.* 2013). We compared the mode of cell death after 24 h treatment with hairpins **1** and **2**, or **Eto** by flow cytometry. We analyzed plasma membrane permeability and phosphatidylserine exposure on the cell surface to determine subpopulations of live, necrotic and apoptotic cells after compound exposure. Plasma membrane permeability was assessed through 7-AAD dye exclusion and phosphatidylserine was visualized by dye-conjugated annexin V binding. We found that the non-treated control and treatment with hairpin **2** at 24 h resulted in little cell death, but treatment with **1** or **Eto** caused significant cell death (Fig. 4c). Remarkably, there was a distinct and substantial population of cells treated with **1** that lost plasma membrane integrity without phosphatidylserine exposure, indicative of a necrotic cell death. We observed a continuous population from the upper left to the upper right quadrant in cells treated with polyamide **1**, suggestive of progressive phosphatidylserine exposure in permeabilized cells. The upper right quadrant in all treatment conditions is secondary necrosis as there are no phagocytes to remove apoptotic or necrotic bodies. There are nearly no cells in the etoposide-treated condition that lie in the upper left quadrant, reflective of a canonical apoptotic cell death with intact membranes displaying phosphatidylserine. The anthracyclins could not be used in this assay due to their inherent fluorescence. We further did a time course of the necrotic effect of polyamide **1** at 12, 24 and 48 h and found that the cells began permeabilization as early as 12 h, and underwent further necrosis by 48 h (Fig. 4d). These assays together suggest polyamide **1** preferentially tracks Raji cells toward a slow, necrotic-type cell death even though the cells are capable of apoptosis.

### Polyamide 1 triggers the release of DAMPs

**Fig. 5:**
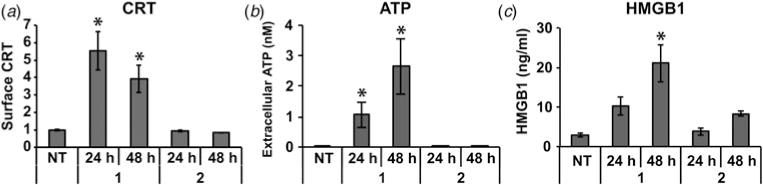
Immunogenic signaling is triggered by polyamide **1** treatment. (*a*) CRT was measured by flow cytometry after 24 and 48 h treatment with **1** or **2** at 25 μM. (*b*) Extracellular ATP was measured by a bioluminescence assay after the same treatment. (*c*) HMGB1 in the supernatant was measured by ELISA after treatment as in (*a*) and (*b*). CRT flow cytometry and HMGB1 ELISA were measured in triplicate from at least two independent experiments. ATP was measured in quadruplicate in three independent experiments. Error bars show standard deviations and asterisks indicate statistically significant increases (two-tailed Student’s *t* test, *p* < 0.05) compared with non-treatment.

The inflammatory nature of necrotic cell death can be attributed in part to the release of immune-activating DAMPs (Rubartelli & Lotze, 2007; Kaczmarek *et al.* 2013). We measured levels of CRT, ATP and HMGB1 after polyamide **1** treatment as these DAMPs have been described as key factors in a spatiotemporal code of immunogenicity (Zitvogel *et al.* 2010; Kepp *et al.* 2011). We included polyamide **2** as an in-class control because **2** has been extensively studied in other cancer models (Yang *et al.* 2013). We measured CRT by flow cytometry after 24 and 48 h of compound exposure at 25 μM and observed a decreasing trend for surface CRT (Fig. 5a), consistent with reports that CRT is an early response signal (Kepp *et al.* 2011). Even at the 25 μM concentration of polyamide **2**, we detected no increase of CRT after 24 h treatment. Extracellullar ATP was measured with a bioluminescence assay of the supernatant after the same treatment regime (Fig. 5b). The ATP detected in the media after treatment with hairpin **1** was greatly increased over the non-treated condition. We next analyzed the release of HMGB1 by running a sandwich ELISA for HMGB1 in the supernatant. After treatment for 24 or 48 h with **1**, a significant increase in excreted HMGB1 was detected in the media (Fig. 5c). The polyamide **1**-mediated release of CRT, ATP and HMGB1 in this temporal pattern is consistent with immunogenic signaling described in the literature.

### Polyamide 1 treated Raji cells *are* subject to phagocytosis

**Fig. 6:**
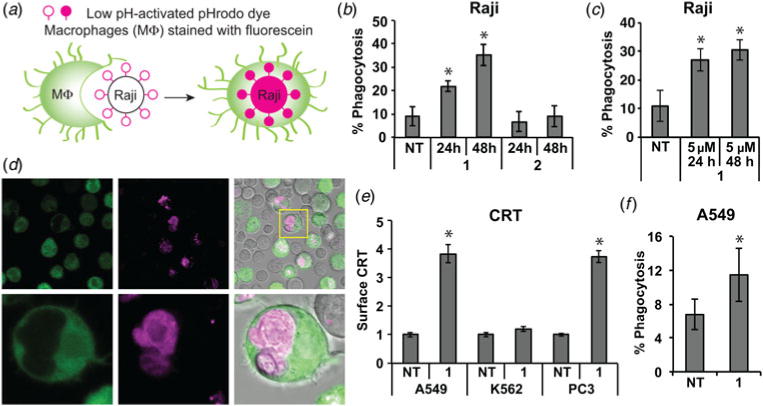
Treatment of cells with **1** increases phagocytosis by human macrophages. (*a*) Schematic diagram of fluorescence-revealed phagocytosis using the pH-sensitive pHrodo dye. (*b*) Raji cells treated with the indicated compound at 25 μM for 24 or 48 h were incubated with human macrophages for 2.5 h. Fluorescein+ cells were assessed by flow cytometry for double-positive cells to determine % phagocytosis. (*c*) Raji cells were treated with a lower dose of 5 μM of **1** for 24 and 48 h and assessed for phagocytosis by macrophages in the same manner as in (*b*). (*d*) Fluorescent images of cells prepared as in (B) are shown. Human macrophages are marked green, free Raji cells are gray in the brightfield composite image, and magenta-colored bodies inside macrophages are phagocytosed Raji cells with activated pHrodo. Yellow box in top row is magnified in bottom row images. (*e*) The cell lines A549, K562 and PC3 were treated with polyamide **1** for 24 h and screened by flow cytometry for surface CRT. Measurements and standard deviations are in technical triplicate and biological duplicate. (*f*) A549 lung carcinoma cells treated with **1** for 24 h was subjected to human macrophages as described above. Graphs show mean and standard deviation from three independent experiments for (*b*), (*c*), (e) and (*f*). Asterisks indicate statistically significant increases (Student’s two-tailed *t* test, *p* < 0.05) compared with the non-treated condition. Images in (*d*) are representative of two independent experiments.

Necrotic cell death with an abundance of externalized CRT and other released immunogenic DAMPs should increase phagocytosis. We obtained human peripheral blood macrophages and incubated them for 2.5 h with Raji cells treated with **1** or **2** at 25 μM for 24 or 48 h. The Raji cells’ plasma membrane was covalently decorated with a pH-sensitive dye (pHrodo) that is activated when engulfed. Macrophages were marked with a cell-permeable fluorescein dye (Fig. 6a). Macrophages that phagocytosed Raji cells are double positive for fluorescein and activated pHrodo dye. In each experiment, 1000 macrophages were analyzed by flow cytometry per condition. Compared with non-treatment, we found that a greater percentage of macrophages incubated with polyamide **1**-treated Raji cells were double positive, and more so at 48 than 24 h (Fig. 6b, SI Fig. 7). We saw little change when macrophages were incubated with cells treated with **2**. This indicates macrophages phagocytosed Raji cells more efficiently after treatment with polyamide **1**. We also tested the lower concentration of 5 μM of **1** used in the library screening assay, anticipating that immunogenicity may be a threshold effect and that 5 μM may be a more reasonable *in vivo* concentration. We found that the treatment of Raji cells with this lower concentration still increased the percentage of phagocytosis by macrophages (Fig. 6c). We then verified the phagocytosis assay results with confocal microscopy to ensure that Raji cells were indeed phagocytosed rather than externally adherent (Fig. 6d). The images show that a fraction of macrophages have internalized Raji cells, which fluoresce with the activated pHrodo dye. The brightfield composite image shows free Raji cells in grey which are not engulfed by macrophages and lack a robust pHrodo signal. The bottom row shows a magnification of a macrophage after phagocytosis of a large Raji cell body.

### Polyamide 1 triggers CRT and phagocytosis in other cancer cell lines

We next explored whether this effect might be specific to the Raji B-cell lymphoma model or more general in other cancer cell lines. We screened the A549 lung adenocarcinoma cell line, K562 B-cell leukemia cell line and the PC3 prostate cancer cell line for CRT translocation after treatment with **1**. We observed approximately fourfold increase in CRT surface expression in the A549 and PC3 cell lines (Fig. 6e). Surprisingly, we did not observe an increase in the K562 B-cell leukemia cell line (Fig. 6e) though they are closest in lineage with the Raji B cells. We then tested the A549 cell line in the phagocytosis assay after 24 h treatment with 25 μM polyamide **1**. Even though the adherent A549 cell line presents a more difficult target for macrophages, as compared with non-adherent Raji cells, we measured an observable increase in A549 phagocytosis after treatment with polyamide **1** (Fig. 6f). These results show the effects of polyamide **1** extend beyond the Raji cell line to cells of different lineages but are not completely general.

## Discussion

In this study, we have discovered a Py–Im polyamide capable of triggering the release of immunogenic signals in a necrotic-type cell death. Treatment with polyamide **1** increased the externalization of CRT, HMGB1 and ATP, which have been characterized to be key signals of immunogenicity. We further observed that polyamide **1** causes permeabilization of the plasma membrane in a subpopulation of treated Raji cells, which grows from 12 to 48 h. This occurs even as the apoptotic cell death pathway remains competent, as demonstrated by the distribution profile observed after **Eto** treatment of Raji cells. We conclude that polyamide **1** preferentially triggers a necrotic-type, likely highly immunogenic cell death, an effect we are not aware has precedent with a small molecule synthetic ligand.

Phagocytosis by antigen-presenting cells is a critical step in a chain of events toward leveraging the specificity and power of the immune system toward attacking the offending target. We saw increased phagocytosis of polyamide **1**- treated cells by macrophages, as would be expected upon the death of Raji cells with externalized pro-phagocytic CRT. The observed phagocytosis and inflammatory DAMPs released into the tumor microenvironment makes plausible that the immune system would become primed toward antigens of Raji cells. Reports have shown engulfment by macrophages can activate an effective anti-cancer *T* cell response (Tseng *et al.* 2013). The application of polyamide **1** on cancer in the context of a full immune system remains to be explored.

That the DNA sequence preference of **1** is identical with that of human telomeres was indicated by the pairing rules and confirmed by Bind-n-Seq. Whether the mechanism of action for polyamide **1** is through its sequence-specific DNA binding capacity remains an open question. Though polyamides have generally been characterized to act in a DNA-binding mode, we cannot exclude that polyamide **1** may act as an aptamer binding to unknown target proteins. The translocation of CRT after polyamide **1** treatment was observed in multiple, though not all, cell lines and suggests there is a conserved pathway that leads to this phenotype that is retained in many cancer cell lines. We expect the elucidation of the mechanism of action will reveal important signaling networks involved in immunosurveillance of cancer cells.

## Conclusion

The effects of polyamide **1** are compelling because immunogenic cancer cell death is cleared in the natural setting and thus difficult to observe. Further study may reveal tumor suppressive signaling pathways that can be exploited to extrinsically control cancer even as cell-intrinsic mechanisms fail. The failure of anthracyclins to elicit CRT in Raji cells when it has previously been shown to be effective in CT26 murine colon cancer cells (Obeid *et al.* 2007) underscores the heterogeneity of cancers. As cancers have heterogeneous mechanisms for evading elimination by the immune system, a corresponding diversity of immunogenicity agents will be important for both shedding light on new biology and developing immuno-oncologic therapies.

## Supplementary Material

S1
