# Supplementary material for: A sequence-specific DNA binding small molecule triggers the release of immunogenic signals and phagocytosis in a model of B-cell lymphoma
Source: Q Rev Biophys. Author manuscript; Available in PMC 2016 Feb 5. (PMC4743504; doi:10.1017/S0033583515000104)
Supplement: S1 [file NIHMS753947-supplement-S1.pdf]

`

A sequence-specific DNA binding small molecule triggers the release of immunogenic signals and phagocytosis in a model of B-cell lymphoma.

*JeenJoo S. Kang and Peter B. Dervan**

Division of Chemistry and Chemical Engineering, California Institute of Technology, Pasadena, CA 91125, USA.

Supplemental Information: Figures and Tables

1) Full chemical structures of polyamides
2) Polyamide mass spectrometry data
3) CRT histogram of dose-dependence and controls.
4) Table of motifs and associated e-values for Bind-n-Seq of biotin-conjugate polyamide **1b**.
5) Table of cell death categorization by flow cytometry after different treatments for 24 hours.
6) Table of time course of cell death categorization after treatment with **1**.
7) Representative flow cytometry data for phagocytosis assay of Raji cells by peripheral blood macrophages after treatment with polyamide **1**.

(*a*)

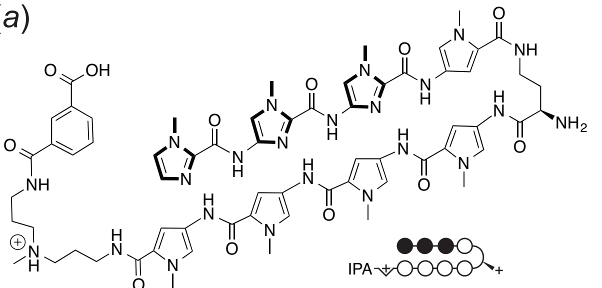

1

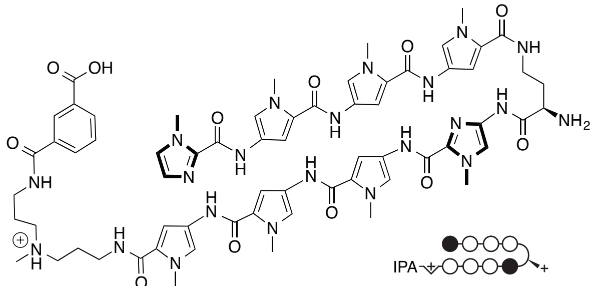

2

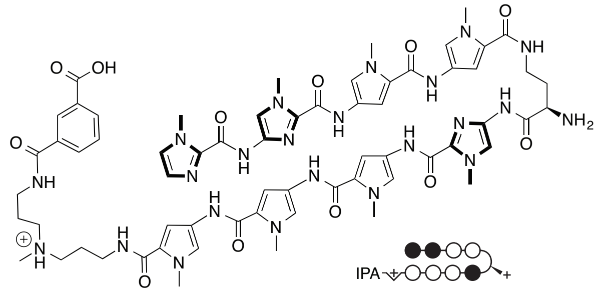

3

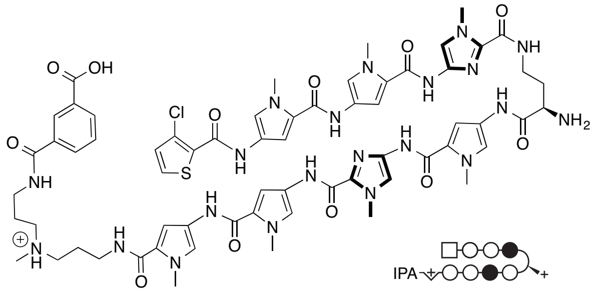

4

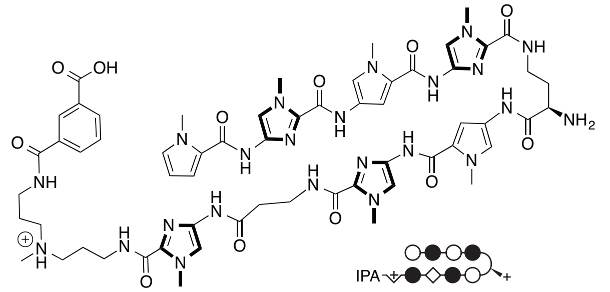

5

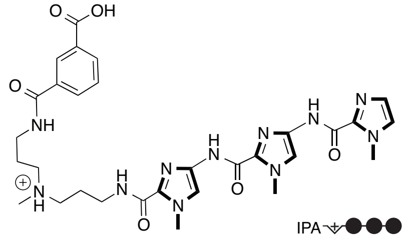

6

(*b*)

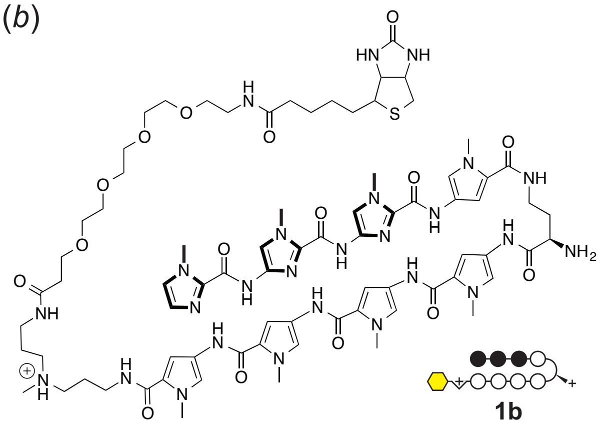

1b

SI Figure 1. Full structures of Py-Im polyamides. (a) Polyamides in CRT screen. (b) Biotin-conjugate of polyamide **1**.

Table S2. Mass spectrometry (MALDI-TOF) for Py-Im polyamides.

**Polyamide**	**Molecular formula**	**[Mass + H]**	**Found Mass**
ImImImPy-(R)$^{\alpha\text{-NH2}}$γ-PyPyPyPy-(+)-IPA (**1**)	$C_{64}H_{76}N_{23}O_{12}^{+}$	1358.6	1358.5
ImPyPyPy-(R)$^{\alpha\text{-NH2}}$γ-ImPyPyPy-(+)-IPA (**2**)	$C_{65}H_{77}N_{22}O_{12}^{+}$	1357.6	1357.1
ImImPyPy-(R)$^{\alpha\text{-NH2}}$γ-ImPyPyPy-(+)-IPA (**3**)	$C_{64}H_{76}N_{23}O_{12}^{+}$	1358.6	1358.0
CtPyPyIm-(R)$^{\alpha\text{-NH2}}$γ-PyImPyPy-(+)-IPA (**4**)	$C_{64}H_{73}ClN_{21}O_{12}S^{+}$	1394.5	1394.1
PyImPyIm-(R)$^{\alpha\text{-NH2}}$γ-PyImβIm-(+)-IPA (**5**)	$C_{60}H_{74}N_{23}O_{12}^{+}$	1308.6	1309.0
ImImIm-(+)-IPA (**6**)	$C_{30}H_{38}N_{11}O_{6}^{+}$	648.3	648.8
ImImImPy-(R)$^{\alpha\text{-NH2}}$γ-PyPyPyPy-(+)-4PEG-Biotin (**1b**)	$C_{77}H_{107}N_{26}O_{16}S^{+}$	1683.8	1683.1

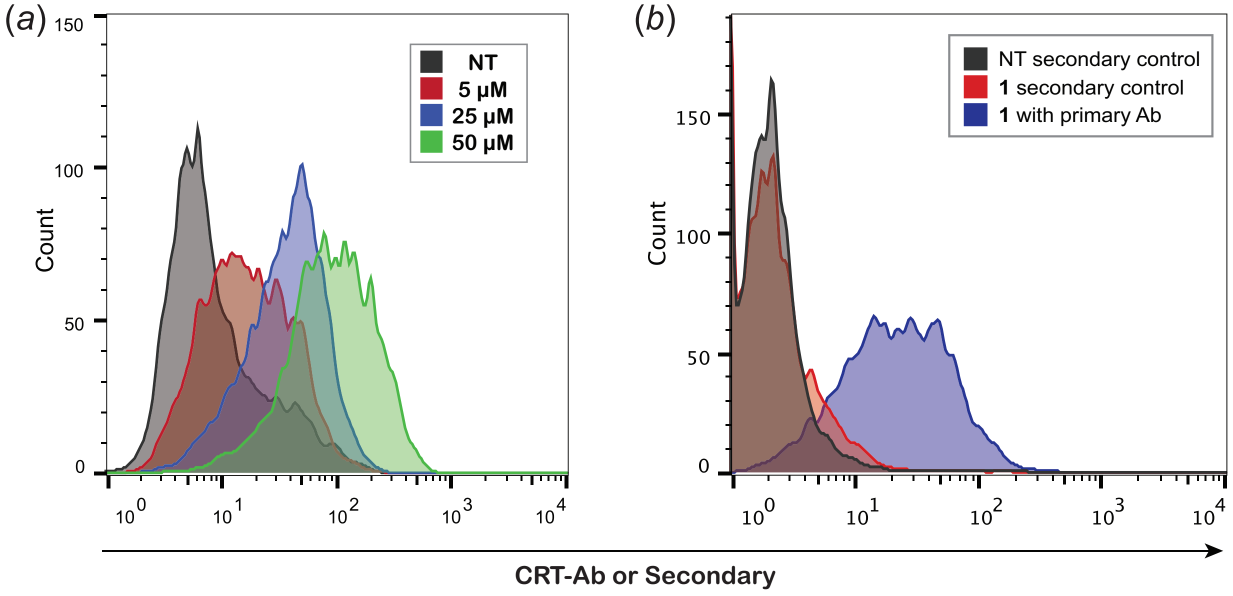

SI Figure 3. CRT dose-dependence data and controls. (a) Representative histograph of CRT dose-dependence after treatment with polyamide **1** at 5, 25, and 50 μM, as measured by flow cytometry. (b) Antibody specificity control: secondary antibody with or without primary antibody after treatment with **1**.

SI Table 4. Table of top motifs and associated e-values for three Bind-n-Seq analyses of biotin-conjugate polyamide **1b**.

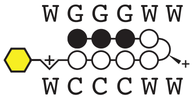

1b

Concentration	Primary motif	E-value	Secondary motif	E-value
250 nM	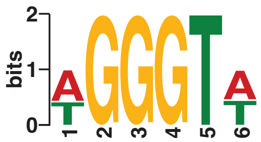	2.6e-5582	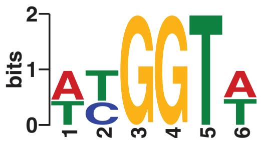	4.5e-207
25 nM	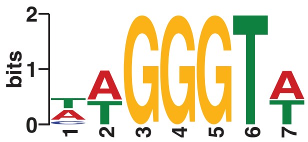	7.3e-509	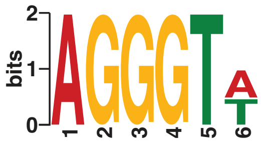	1.1e-028
25 nM	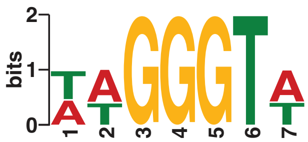	3.9e-129	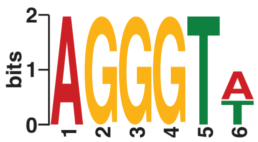	9.1e-008

SI Table 5. Table of cell death flow cytometry after different treatments for 24 hours.

	**Live**	**Necrotic**	**Apoptotic**	**2º Necrotic**
**NT**	86.9 (±1.6)	2.0 (±0.4)	5.8 (±1.3)	5.3 (±0.3)
**1**	57.0 (±2.2)	11.9 (±1.1)	12.8 (±1.2)	18.4 (±1.2)
**2**	88.1 (±1.0)	2.3 (±0.3)	5.2 (±0.9)	4.5 (±0.2)
**Eto**	26.7 (±0.7)	5.3 (±0.2)	32.7 (±1.1)	35.3 (±1.0)

SI Table 6. Table of cell death flow cytometry after treatment with **1** over time.

	**Live**	**Necrotic**	**Apoptotic**	**2º Necrotic**
**NT**	95.7 (±0.5)	1.7 (±0.4)	1.1 (±0.1)	1.5 (±0.1)
**12 h**	47.5 (±1.1)	19.6 (±2.0)	14.4 (±1.1)	18.5 (±0.5)
**24 h**	59.7 (±4.8)	15.6 (±1.0)	11.8 (±1.0)	12.9 (±3.0)
**48 h**	79.4 (±1.5)	7.7 (±1.1)	6.7 (±0.3)	6.2 (±0.4)

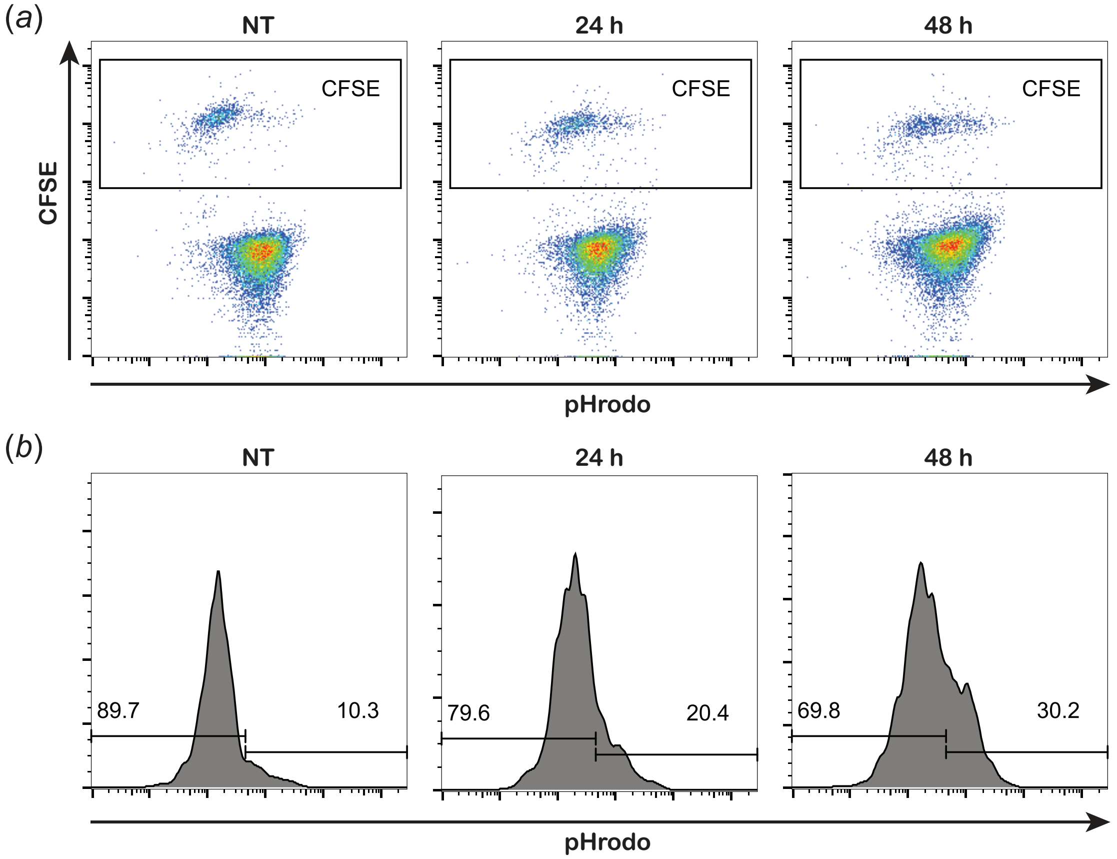

SI Figure 7. Representative flow cytometry data for phagocytosis assay of Raji cells by peripheral blood macrophages after treatment with polyamide **1**. (a) Raw data showing gate of CFSE+ population, peripheral blood macrophages. (b) Histograph of pHrodo dye in gated macrophage population of (a).
